# Selections from the Surgical Clinics, Medical Department of Niagara University

**Published:** 1884-05

**Authors:** Frank Hamilton Potter


### Selections from the Surgical Clinics, Medical Department of Niagara University—Session of 1883–4.

Reported by Frank Hamilton Potter, M. D.

The following cases are selected from those presented at the regular surgical clinics of Prof. W. S. Tremaine, at the Hospital of the Sisters of Charity, Buffalo, during the winter of 1883-4.

These clinics were given primarily for the students of the medical department of the University of Niagara, but they were generally attended by others not connected with the University—students and medical gentlemen of the city interested in the science and art of surgery.

In the report it is intended to give merely the history in outline of each case, desiring in this way to show the great variety of surgical injuries and diseases presented for study, and the uniformly good results obtained. The etiology, pathology, and treatment are thoroughly explained to the students as the patients are brought before them, and whenever the opportunity offers they are invited to examine the case and make a diagnosis, and are thus taught in a practical manner the art of physical diagnosis. This method of bedside instruction is one of the features of the University, the object being to make the students perfectly familiar with the best manner of conducting an examination, and to teach them how to apply in actual practice the principles taught in the lecture room. During the surgical operations they are frequently asked to render assistance, and thus, at an early period of their studies, become familiar with the many details attending any surgical proceeding.

Before presenting the cases it may be proper to explain just what is meant by the term "antiseptic surgery," as made use of in this report. Dr. Tremaine teaches the principles of aseptic surgery as founded upon the latest scientific investigations. Listerism is not carried to the extent advocated by its author, but in the treatment of wounds and surgical injuries the following principles are insisted upon:

I. The thorough cleansing of all wounds.

II. The *entire* arrest of hemorrhage.

III. Complete drainage, particularly of all deep-seated wounds.

IV. The use of antiseptic agents, such as bichloride of mercury, iodoform, carbolic acid, alcohol, etc., in dressing all wounds, in disinfecting the instruments, the hands of the operator and assistants, and the parts of the patient to be operated upon.

V. Protection of the wound from any deleterious external influences.

VI. The use of catgut, which has been rendered antiseptics for sutures and ligatures wherever possible, and when from the nature of the wound this cannot be used, the substitution of gold and silver wire.

VII. The use of antiseptic dry absorbent dressings, such as borated cotton, marine lint, jute, sublimated peat, etc.

VIII. The dressings are not changed unless there is some particular indication for so doing. This latter point is especially insisted upon, and with the most pleasing results. If the above principles are thoroughly applied, it is seldom necessary to disturb the wound until healing is completed. Of course, when any unfavorable indication is observed, either in the condition of the patient or the wound, the dressings are removed at once.

The care necessary in the application of these principles to the treatment of wounds is amply rewarded, as the results in the following cases will show:

*Case* 1. James Morelli, Italian; age, 60. This man was injured upon the railroad, sustaining a compound fracture and dislocation of the left ankle. He was not seen by Dr. Tremaine until three days after injury, when the leg was found to be in a condition of intense cellulitis, with commencing gangrene over the internal malleolus. Symptoms of septicæmia. Amputation of leg at union of proximal and middle third; antiseptic dressing and union by first intention, the patient being around the ward within ten days. This man could not speak a word of

English, and could with difficulty be made to understand what was required of him. He was exceedingly filthy in his habits, urinating in bed, etc. A large bed-sore developed over the sacrum, which retarded his convalescence. It seems he was under the impression that his leg would grow again, and when he became satisfied from conversation with some of his countrymen that this could not occur he was greatly depressed and refused to leave his bed. Other bed-sores formed, and symptoms of pyæmia developed, from which he finally died. The septic infection was probably produced through the bed-sores.

*Case* 2. T. B., American; age, 22. Two days previous to admission his right hand was caught between the bumpers on freight cars and severely contused. The skin was not broken and the injury did not extend above the wrist. He refused to allow the attending surgeon to treat it in the proper manner, and was finally persuaded to enter the hospital. Upon admission, the entire hand was found to be gangrenous, and the fore-arm severely inflamed, the cellulitis extending to the elbow. There were also symptoms of septicæmia. The arm was amputated at the juncture of the middle and lower third, and antiseptic dressings applied. Slight suppuration occurred in this case, but he made a good recovery, being around the ward in two weeks.

*Case* 3. George B., American; age, 26; epithelioma on left side of nose, four centimeters in length and two centimeters in width. This was removed by elliptical incisions, the wound sprinkled with iodoform and dressed with marine lent. Patient discharged in one week, the wound having healed without change of dressing. This case was interesting from the fact that the father of the young man presented himself at the clinic with the same disease, involving about the same amount of tissue in the left breast. This was also removed, but recovery was not as rapid as in the son's case. He was quite unruly and succeeded in tearing out the stitches. The wound was then allowed to heal by granulation, it being dressed every few days with iodoform and marine lint. Recovery occurred in four weeks.

*Case* 4. C. M., Irish, age 25, while working upon the Cantilever bridge, at Niagara Falls, lost his balance and fell a distance of sixty feet, striking upon the rocks below. Besides several contusions of the soft parts, he sustained a simple fracture of the tibia and fibula of left leg at the middle third. For the first week the leg was dressed with lateral paste-board splints. These were then removed and the Bavarian apparatus applied. Patient discharged fully recovered at the end of six weeks.

*Case* 5. G. S., English; age, 11½; admitted to the hospital with the right arm, at the juncture of the proximal and middle third of the humerus, severed by railroad car passing over it. The remaining part of the arm was severely contused and lacerated. Amputation was performed at the shoulder joint. All bleeding points were secured and the flaps accurately adjusted. From the condition of the soft parts it was considered probable that sloughing of the flaps would occur, and the injuries of the vessels justified an apprehension of secondary hemorrhage. Both these accidents happened. Hemorrhage was detected on the second day after the operation, and again on the eighth day. In both instances the blood came with great force, and was apparently from the axillary artery. The bleeding was promptly controlled by the resident internes. The sloughing of the flaps delayed convalescence, that part of the wound healing by granulation. The boy rallied well from the injuries and from the operation, the temperature never rising above 101° F. Notwithstanding the interference of these various accidents with the healing process, recovery was rapid, the patient leaving the hospital at the end of eight weeks. The antiseptic method was carefully used in this case; as soon as the sloughing occurred and pus appeared, dry absorbent dressings were applied, and these were changed every second or third day, according to the necessity. The appropriate constitutional treatment was employed.

*Case* 6. M. J., Irish; age, 40; admitted to hospital with extensive disease of elbow joint of left arm of three years' standing. There were various sinuses opening into the joint, through which pus was discharging. The tissues were swollen and of a reddish color, showing the disease was not confined to the bones. The patient stated that three years before, without accountable cause, an abscess formed, which was opened, and from which a quantity of sanious pus was discharged. This abscess communicated with the cavity of the joint. Patient was etherized on March 10th, and an exploratory incision made with the view of excising the elbow joint. It was found, however, that the disease had progressed to such a degree, and disorganization was so extensive, that amputation rather than excision would be justifiable. This was accordingly performed at the juncture of the proximal and middle third of the arm. Even then an osteo-myelitis was found to extend above the point of amputation. The antiseptic method was carefully used in this case. The patient rallied well from the operation, the temperature and pulse remaining normal during the entire course of treatment. There was no suppuration, the wound healing by the first intention. Four weeks after the operation the wound had been dressed but twice.

*Case* 7. E. G., German; age, 28. His left arm was caught between bumpers on railway carriages, causing a compound comminuted fracture of the elbow joint. The external wound extended from the external condyle of the humerus upwards and outwards a distance of nine centimeters. The brachial artery and median nerve were exposed so that they could be picked up between the fingers. A counter-opening was made on the posterior surface of the arm, and the comminuted bone removed. This consisted of the head of the radius, the external condyle of the humerus and part of the olecranon process of the ulna. The wound was then irrigated with a solution of mercuric bichloride (1 part to 1,000), a drainage tube passed through from the anterior to the posterior wound, the fore-arm placed at an angle of 90°, with the arm and the elbow surrounded with bags of peat which had been soaked in the antiseptic solution. These bags were held in place by bandages. The third day after injury the pulse was 108, the temperature 101° F. The next day both pulse and temperature were normal. The temperature did not rise again above normal, with one exception. On the eighth day the patient had a diarrhœa, with a temperature of 100° F. The diarrhœa was controlled by small doses of Dover's powder, and convalescence was not again interrupted. There was no suppuration. The wounds were dressed but twice during the three weeks following the injury. At this time the patient was walking around the ward, his arm supported by a splint placed on the under surface. The external wounds had nearly healed.

(*To be Continued.*)

